# Network Design and Quality Checks in Automatic Orientation of Close-Range Photogrammetric Blocks

**DOI:** 10.3390/s150407985

**Published:** 2015-04-03

**Authors:** Elisa Dall’Asta, Klaus Thoeni, Marina Santise, Gianfranco Forlani, Anna Giacomini, Riccardo Roncella

**Affiliations:** 1 Department of Civil, Environmental, Land Management Engineering and Architecture, University of Parma, via Parco Area delle Scienze, 181/a, 43124 Parma, Italy; E-Mails: elisa.dallasta@studenti.unipr.it (E.D.A.); marina.santise@studenti.unipr.it (M.S.); gianfranco.forlani@unipr.it (G.F.); riccardo.roncella@unipr.it (R.R.); 2 Centre for Geotechnical and Materials Modelling, The University of Newcastle, 2308 Callaghan, Australia; E-Mail: anna.giacomini@newcastle.edu.au

**Keywords:** photogrammetry, structure-from-motion (SfM), 3D model, accuracy, laboratory experiment, *in situ* experiment, ground control, image metrology

## Abstract

Due to the recent improvements of automatic measurement procedures in photogrammetry, multi-view 3D reconstruction technologies are becoming a favourite survey tool. Rapidly widening structure-from-motion (SfM) software packages offer significantly easier image processing workflows than traditional photogrammetry packages. However, while most orientation and surface reconstruction strategies will almost always succeed in any given task, estimating the quality of the result is, to some extent, still an open issue. An assessment of the precision and reliability of block orientation is necessary and should be included in every processing pipeline. Such a need was clearly felt from the results of close-range photogrammetric surveys of *in situ* full-scale and laboratory-scale experiments. In order to study the impact of the block control and the camera network design on the block orientation accuracy, a series of Monte Carlo simulations was performed. Two image block configurations were investigated: a single pseudo-normal strip and a circular highly-convergent block. The influence of surveying and data processing choices, such as the number and accuracy of the ground control points, autofocus and camera calibration was investigated. The research highlights the most significant aspects and processes to be taken into account for adequate *in situ* and laboratory surveys, when modern SfM software packages are used, and evaluates their effect on the quality of the results of the surface reconstruction.

## 1. Introduction

Computer vision (CV) has significantly contributed to the improvement of image-based object reconstruction, most notably to sensor calibration and orientation [1,2]. While photogrammetry focuses primarily on image metrology, *i.e.*, on the precision and reliability of the results [3], CV aims at the orientation of large blocks (sometimes thousands of images) taken with un-calibrated cameras mainly for 3D visualisation and image browsing [4]. Nevertheless, CV-based techniques and software packages are more and more being used for engineering [5,6], geology [7], geoscience [8,9,10] and cultural heritage [11].

The effectiveness of automatic image orientation algorithms, such as structure-from-motion (SfM), is leading to profound changes in the field of traditional photogrammetry. Although the goal of the complete automation of the photogrammetric pipeline is still far away (and probably not fully required), it removes one of the most critical stages in the procedure. Automated image orientation is a very welcome step, especially for non-expert users. Indeed, SfM even allows for the successful orientation of complex and unconventional blocks [12]. Nevertheless, estimating the quality of the results in terms of exterior orientation (EO) parameters and object point accuracy seems still to be an open issue, especially when self-calibration is applied [13].

Several commercial software packages allow the automatic calibration of the interior orientation (IO) parameters to be performed during a bundle adjustment. The robustness achieved by the state-of-the-art SfM algorithms in the orientation of images taken with cameras with unknown IO parameters may lead the non-expert user to underestimate the importance of camera calibration. In some cases, inaccurate camera calibration may significantly affect the orientation results. For instance, IO and EO parameters can be strongly correlated, and this might lead to systematic deformations of the reconstructed model [14]. The high number of image observations used by SfM procedures does not in itself guarantee the correctness of the result and the required quality level. Metric performances of SfM should therefore be more thoroughly investigated in order to extend the use of these techniques to image metrology [15].

Furthermore, the high automation level of SfM software packages can hide important aspects, such as the influence of camera autofocus and the consequences of an inaccurate pre- or self-calibration procedure of the optical system [14]. It seems more reasonable and also more appropriate than in standard photogrammetric tasks to check the restitution error for object coordinates, though it involves also the image measurement accuracy, the IO accuracy and the accuracy of check points or ground control points (GCP).

In the so-called normal case, the precision of stereo restitution in photogrammetry is often used as a reference for a preliminary survey design [14]. In general, the error propagation relates the image measurement precision to the object point precision. Assuming known IO and EO parameters, the precision along the optical/depth axis $\sigma_{Z}$ (usually worst compared with the other directions) can be defined as:

(1) $$\sigma_{Z}=\frac{Z^{2}}{cB}\sigma_{p_{\xi }}$$

It depends on the distance Z to the object, the base length B, the focal length c and the precision of the image measurements $\sigma_{p_{\xi }}$. The image measurement accuracy depends primarily on the accuracy of feature extraction and feature matching, where sub-pixel accuracy can generally be achieved [16]. For cases where the object can be entirely pictured in a single frame, a very common case in close-range, a convergent camera network configuration is often used. For such cases, Fraser [17] suggested to evaluate the standard error σ of the *x*, *y* and *z* object coordinates as:

(2) $$\sigma =\frac{qZ}{c\sqrt{k}}\sigma_{p_{\xi }}$$

where *k* corresponds to the average number of exposures at each station and *q* is a design factor expressing the strength of the camera network, basically dependent on the angles between intersecting homologous rays. The precision is the same in all directions if the intersections between the rays are nearly 90°. On the one hand, convergent poses are preferable, since higher intersecting angles can be obtained. On the other hand, higher perspective changes can make the matching process less accurate if automatic image matching techniques are used. In other words, $\sigma_{p_{\xi }}$ in Equation (2) tends to grow as the relative angles between the pictures become higher, hence reducing at some point the precision of the estimated object coordinates. Convergent networks are also used in circular blocks when the image sequence runs around the object maintaining an approximate constant distance (and thus, a constant image scale) from the surface of the object. Equations (1) and (2) give a rough estimation of the expected precision, but several parameters have to be known *a priori*. External checks relying on independent information should provide a direct or indirect evaluation of the results.

This paper discusses some of the fundamental aspects for precision and quality assessment of automatic oriented image blocks usually used for multi-view 3D reconstruction. To the authors’ best knowledge, this is the first time that a study on the analysis and identification of critical configurations affecting SfM projects has been presented. The use of modern CV algorithm tools (commercial and open source) gives the impression that 3D modelling from images is easy. However, poor results can be obtained if the basic requirements of photogrammetric block design are not taken into consideration. The paper deals with this topic, showing strategies, considerations and the possible solutions. Starting from some experimental examples, the study is completed with a series of Monte Carlo (MC) simulations developed to pinpoint problems that can affect the quality and the accuracy of the 3D reconstructed scenes. Firstly, the results of photogrammetric surveys of a berm (pile of soil and boulders of rock) conducted at two different scales are presented (Section 2). The first survey was performed *in situ* at full scale and resulted in a pseudo-normal image block. GCP could only be positioned at the extremities of the object. The second survey was performed in the laboratory at a reduced scale. The entire object was visible in a single frame, and this resulted in a circular image block. In addition, GCP were placed all around the object. Secondly, a series of MC simulations based on the previously discussed experiments is presented (Section 3). Two virtual objects and camera networks are introduced representing the two real scenarios. In these scenarios, the influence of various parameters on the accuracy and quality of the photogrammetric block is studied. The parameters considered include: camera calibration (pre-calibration *vs.* self-calibration), camera autofocus, number of GCP, location of GCP, accuracy of GCP and number of tie points used in the automated orientation procedure. Finally, conclusions and recommendations are given in Section 4.

## 2. Photogrammetric Surveys

Data from full-scale (1:1) and laboratory-scale (1:50) tests carried out by the University of Newcastle (Australia) are considered in this study. The tests have been conducted within a research project aiming to investigate the hazard related to haul trucks operating in a mine site and identifying the importance safety berms play in preventing haul truck accidents [18]. The berms are made of spoil material collected on site and are currently designed according to a rule of thumb that the height of the berm must be at least half the wheel diameter of the largest vehicle used at the mine site.

The tests involved reversing ultra-class haul trucks, such as the CAT 797, into a berm. Images of the berm were collected before and after the impact in both full-scale and laboratory-scale tests. The images were then used to build the pre-event and the post-event digital surface model (DSM) for each test. By comparing the two DSMs, the deformation of the berm could be assessed [18].

A non-metric off-the-shelf digital camera, the Panasonic Lumix LX5, was used to collect the images in both case studies. The camera uses a 10-Mp high-quality CCD (charge-coupled device) image sensor and a low distortion wide-angle Leica lens. The calibration of the camera was carried out with the software PhotoModeler [19] by adjusting a circular image block of a set of photogrammetric coded markers. The calibration was performed separately for the full-scale test and the laboratory-scale test, since different camera settings and distances to the object were used. 

### 2.1. In Situ Full Scale Tests

**Figure 1:**
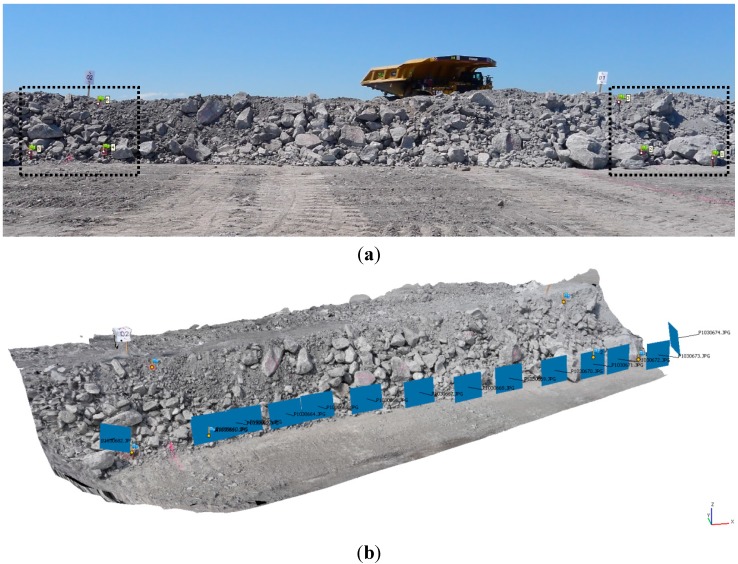
*In situ* full-scale tests: (**a**) typical berm with ground control points (GCP); (**b**) corresponding typical parallel image block.

A series of *in situ* full-scale tests was undertaken on a remedial dumpsite located within an open cut mine in the Upper Hunter Valley, Bulga, NSW, Australia [18]. Several test bays, each about 13–15 m wide, were marked along the berm, and three targets for ground control were installed on each side of each bay. Figure 1a shows a typical test bay setup with the installed GCP. The coordinates of the GCP were measured using a Leica TCRA 1105 reflectorless total station with an angular accuracy of 5'' and a distance accuracy of ±(2 mm + 2 ppm). 

Once ground control was installed, digital still images of the berm were collected by an operator. The images consist of a mainly parallel image block taken from a distance of about 2–3 m by walking parallel along the berm. Figure 1b shows a typical DSM, including the image block adopted for the full-scale tests.

### 2.2. Laboratory Scale Tests

**Figure 2:**
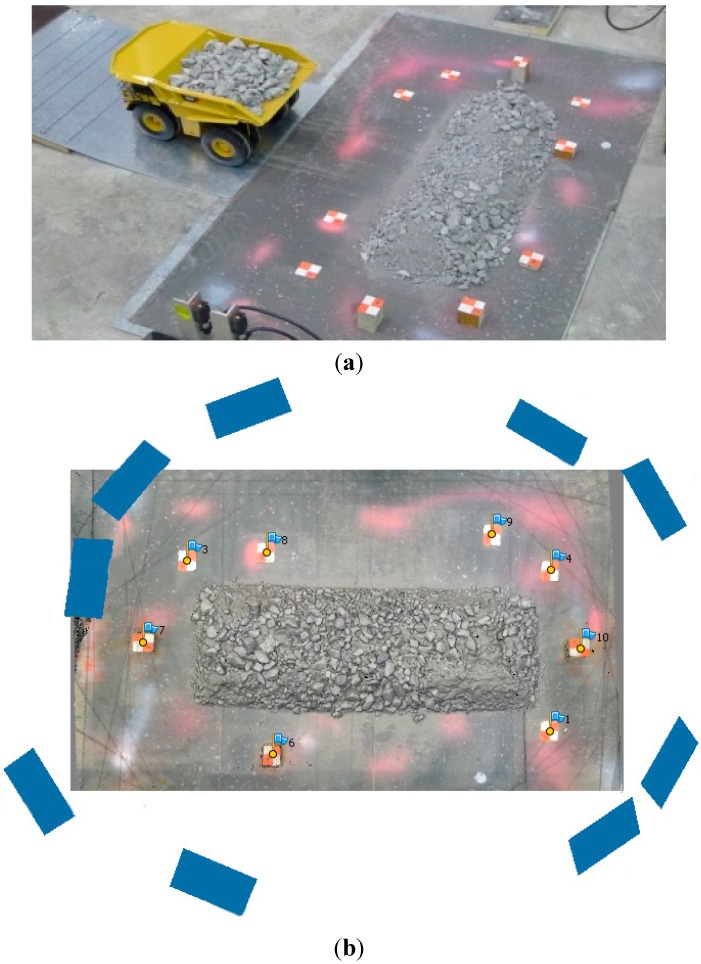
Laboratory-scale tests: (**a**) typical berm with GCP; (**b**) corresponding typical circular image block.

Laboratory tests at a scale of 1:50 were conducted in the Civil Engineering Laboratory of the University of Newcastle, Callaghan, NSW, Australia [18]. The 1:50 scale berm models, constructed with a trapezoidal geometry consistent with the full-scale tests, were built using the same material as used at the mine site. The particle size distribution in the model was appropriately scaled at 1:50 in order to maintain the principle of similitude between full-scale and reduced-scale models. The experimental setup is shown in Figure 2a. The berms were positioned on top of a rigid plate. Targets were placed on the plate, which served as GCP for the photogrammetric survey. The same total station used for the full-scale tests was used to measure the coordinates of the GCP for the laboratory-scale model. However, multiple stations were used to increase the accuracy to about 0.5 mm.

A nearly circular convergent image block of the berm was collected from distances in the range of 1–2 m (Figure 2b). In contrast to the full-scale tests, where each image covered only a small part of the berm, each image collected in the laboratory covered the whole berm. In other words, the image overlap was usually close to 100% for all of the sequences.

### 2.3. Quality Assessment of the Photogrammetric Survey

The orientation of the image blocks and the generation of the DSM were performed using the commercial software package Agisoft PhotoScan [20]. The standard software workflow is as follows: first, it estimates the images orientation and refines the camera calibration parameters (the geometry of the image sequence allows one to estimate a set of IO parameters for each camera, if these are not previously assigned); in the second step, it proceeds to the DSM generation [21]. Being a sort of “black-box” software, few statistics on the SfM and dense matching processes are given. In fact, due to commercial reasons, very little information about the used algorithms is available (some details can be recovered from the PhotoScan User forum). Nevertheless, an analysis of the covariance matrix of the unknown parameters of the bundle block adjustment (BBA) can be performed by exporting the image block and analysing it with a “traditional” photogrammetric software. In this way, the theoretical accuracy of the object point coordinates can be estimated.

In order to verify the reliability and the repeatability of the results, comparisons between several DSMs obtained from the same sequence using different algorithms or different image pairs have been performed for eleven real-scale tests and for eight laboratory-scale tests. Small location errors of the two reconstructed objects (with respect to the reference system defined by the GCP) were minimized by performing an iterative closest point (ICP) alignment procedure. The standard deviations of the distances between the different DSMs have been used as an empirical evaluation of the overall object reconstruction quality.

**Figure 3:**
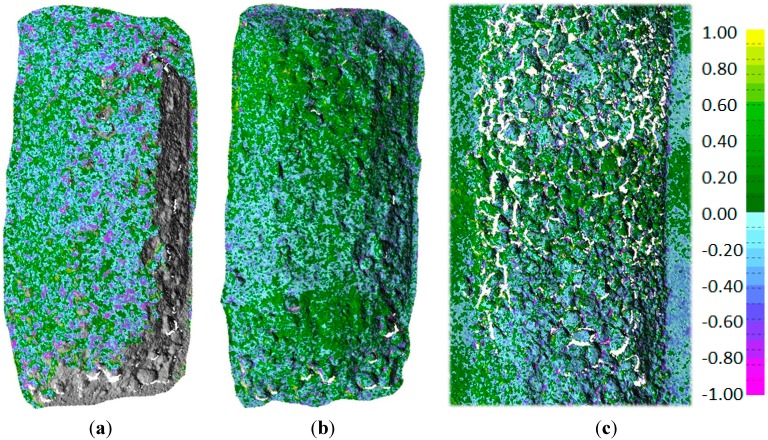
Map of distances (in mm) between the DSM generated with: (**a**) PhotoScan (PS) and Dense Matcher (DM) by using the same image pair; (**b**) PS and DM using the same sequence of three images; (**c**) PS using two different image pairs extracted from an image sequence of a laboratory test.

The analysis was carried out for all eight laboratory-scale tests and all eleven full-scale tests. The results are fairly constant over all models, and hence, only the results of one model are presented in detail. Figure 3 shows the maps of the color-coded differences between two DSMs obtained from one of the laboratory image sequences. Figure 3a,b shows the differences between PhotoScan (PS) and Dense Matcher (DM) [22] on the same image pair and on a sequence of three images of the block, respectively. In both cases, local shape differences are within ±1 mm. The standard deviation of the distances is 0.34 mm for the image pair and 0.29 mm for the sequence of three images. The differences between the two DSMs obtained using the same software (in this case, PS) on two different image pairs are depicted in Figure 3c. The results are of the same order of magnitude as in the previous comparison. Therefore, the differences between the models are more likely to be due to image block characteristics than to the specific DSM generation algorithm used by the two software packages.

The comparisons show that the precision of the DSM generated from the laboratory images varies in the range of 0.4–0.6 mm. However, the expected theoretical precision from Equation (2) considered in the block design and the estimated values obtained by the BBA covariance matrix stated that the object coordinate precision should be in the range of 0.1–0.2 mm. The results are significantly worse (three-times) than the theoretical ones. The same behaviour was found in the full-scale experiments, where the expected precision from Equation (1) was in the order of 2 mm (the image scale in this framework was proportionally much higher than in the laboratory tests), confirmed by the BBA covariance matrix, while the repeatability analysis of the DSM showed values in the range of 2–4 cm (ten-times worse).

To summarize, it was found that, especially at full scale, the theoretical precision expected from the survey design was not matched by the repeatability analysis. In this context, it is important to highlight that Equations (1) and (2) give information about 3D point coordinates of tie points, which are different from the geometric data generated in a DSM. A DSM is generally created by interpolation techniques from points extracted via dense matching. This process generally introduces additional errors. A rigorous approach to check survey compliance with theoretical accuracy should always be performed using well-distributed check points. Thereby, the check points should be measured using an independent surveying technique with a precision at least two- or three-times higher than the one expected in the photogrammetric survey. However, in many experimental setups, support for an additional survey of check points is not provided because of budget or technical constraints. In such cases, a comparison between different DSMs of the same object portion can be used instead. In the latter case, defining this stage as a repeatability analysis is more appropriate and, hence, used in this study.

Nonetheless, the DSM repeatability analysis of the laboratory tests showed an error ten-times worse than the expected theoretical one, and it seems quite questionable to pin this issue on the DSM interpolation only. Especially because the ground sampling distance of the point cloud in the specific studies corresponds to about 2–3-times the cell resolution of the DSM, hence, the interpolation taking place in the DSM generation is likely to smooth matching errors.

It is worth noting that, at reduced laboratory scale, problems due to the accuracy of the GCP, image stability, image focusing, *etc.* (usually not taken into account in the design stage), become more and more important. In addition, the geometric configuration of the full-scale tests can produce high correlations between EO and IO parameters, leading to small residuals in terms of collinearity equations, but perhaps to a significant systematic deformation of the DSM.

For these reasons, and in order to provide a more rigorous and complete theoretical framework of error propagation for all of the various sources that can affect close-range SfM image blocks, a series of MC simulations was conducted.

## 3. Numerical Simulations

The results presented in the previous section raise some issues on the accuracy of the DSM at different scales. The models of Equations (1) and (2) used for block design in the normal and convergent case, respectively, as well as the stochastic error model implemented in the BBA procedure are based on assumptions that seem not to be satisfied in the experiments. The equations indicate that the precision of the 3D point coordinates should scale according to the image scale. Therefore, there should be the same ratio between the repeatability measures and the expected precisions in the two cases. However, the analysis of the DSM differences shows quite clearly that such a ratio is 2–3-times worse than expected for the real-scale tests. 

The error propagation models of Equations (1) and (2) are based on assumptions and simplification that can be easily satisfied (at least approximately) in most aerial photogrammetric blocks, but not necessarily in other scenarios. In fact, the error propagation models assume that the IO parameters and the lens distortion model are exactly known. In addition, they assume that the accuracy and distribution of the GCP effectively prevent systematic block deformations and that the block geometry is designed to limit the correlation between all of the geometric parameters. However, it seems that all of these assumptions are not met in the scenarios presented in Section 2. Even if the general requirements for the berm experiment at both scales are fully satisfied (*i.e.*, the repeatability analysis shows that the discrepancies are lower than the sensitivity required to evaluate the deformation after an impact), a further investigation of the causes that led to an accuracy lower than the expected one was necessary.

Since the BBA covariance matrix analysis did not explain the obtained results, a series of MC numerical simulations were performed in order to assess the influence of several factors that are commonly not considered in image block design (especially by non-expert users) and that could affect the final total error.

Five different scenarios were considered in the MC analyses:

- Case 1 is representative of a weak block geometry (e.g., a single normal-case sequence as in the full-scale experiments), where self-calibration routines may produce unpredictable results.
- Case 2 is representative of a photogrammetric survey of very small objects. In such cases, it can be difficult to keep the shooting distance constant for all of the images and to optimize the depth of focus. Hence, a trade-off between better quality images (using, e.g., the camera autofocus) and a more complex BBA procedure is needed. A decision should be made between using a mean value of IO parameters or self-calibrating every frame (in which case, a very strong correlation between the parameters can be expected if the image block geometry is weak).
- Case 3 considers the influence of the number and location of GCP on the effective image block deformation. This applies to both real and laboratory experiments, where achieving a good number and distribution of GCP is not always practicable.
- Case 4 is intended to evaluate how the accuracy of the GCP in a rigid block (*i.e.*, a block with many and well-distributed GCP) may affect the restitution.
- Case 5 investigates the characteristics of image blocks oriented using a very high number of tie points. This is the case with all modern photogrammetric software packages that implement automated orientation procedures based on SfM algorithms. However, the BBA routines that provide the orientation solution are not always designed to properly weigh the collinearity equations for the tie points with respect to the ones involving GCP.

A .NET experimental framework was developed in order to run the MC simulations. The image block geometry is specified using a fairly simple and intuitive configuration file where the user can describe the block structure (e.g., a single normal strip, a circular block with all of the images targeting a specific point or an area, a hemispherical distribution of camera stations, an unordered distribution of stations, a combination of the above, *etc.*). Different object shapes can be defined procedurally or using a discrete set of 3D points: the points are then projected on the image frame and used as tie-points. Thus, in every simulation sample, the same tie points are used and compared. The user can also specify how the ground control is provided (e.g., using a set of GCP, or using a free net bundle block adjustment [23], or constraining the camera poses and locations, *etc.*). Various software packages address this issue in different ways: CV-oriented packages use the set of GCP to estimate a seven-parameter transformation; others perform a free-net adjustment with additional constraints to define the reference system. Photogrammetric packages usually implement GCP constraints in the BBA. To limit the number of parameters in the MC simulations, this aspect was not taken into consideration. For the first three cases, four GCP on the boundary of the image block were used in order to obtain a similar solution for the GCP block constraints and for the free-net BBA. At the same time, in Case 3, the influence of the GCP network was evaluated by controlling the image block deformation. Finally, the procedure to be used during the BBA (e.g., self-calibration, full-field calibration, *etc.*) can be specified, as well. As far as the stochastic model of the bundle adjustment is concerned, each parameter group may have associated its own covariance matrix and can be considered fixed or variable in the block adjustment.

An automated routine performs the MC simulations iterating over the bundle adjustment procedure and collecting the orientation solution and the estimated object structure (coordinates of tie points) at the end of each iteration. The MC framework can be interfaced with several BBA routines. In particular, the CALGE BBA module [24], a widely tested scientific package, was considered the most versatile and efficient for the variety of block configurations in the different case studies. Nevertheless, other software packages were used and their results compared with those obtained with CALGE. More precisely, all of the test cases were run at least once (*i.e.*, with a single MC iteration) using the Damped Bundle Adjustment Toolbox (DBAT) [25,26], as well as the two commercial software packages, PhotoModeler and PhotoScan.

Table 1 shows a list of the series of MC simulations carried out and summarizes the specific features investigated in each series.

**Table 1:** Summary of Monte Carlo (MC) simulations carried out, including investigated features.

TEST		Case	Sequence			Calibration	GCP
			Type	No. of Photos	Overlap		
Calibration Test	Single Strip	1-A	Normal-case	15	80%	Pre-Calibrated	4
		1-B	Normal-case	15	80%	Self-Calibration	4
	Autofocus	2-A	Convergent Circular	8	80%	Pre-Calibrated	4
		2-B	Convergent Circular	8	80%	Self-Calibration	4
GCP Test	Number of GCP	3-A, B, C	Normal-case	20	60%	Pre-Calibrated	4, 8, 16
		3-D	Normal-case	20	80%	Pre-Calibrated	4
	GCP Quality	4	Convergent Circular	8	80%	Pre-Calibrated	8
	GCP to tie points ratio	5	Convergent Circular	8	80%	Pre-Calibrated	8

**Figure 4:**
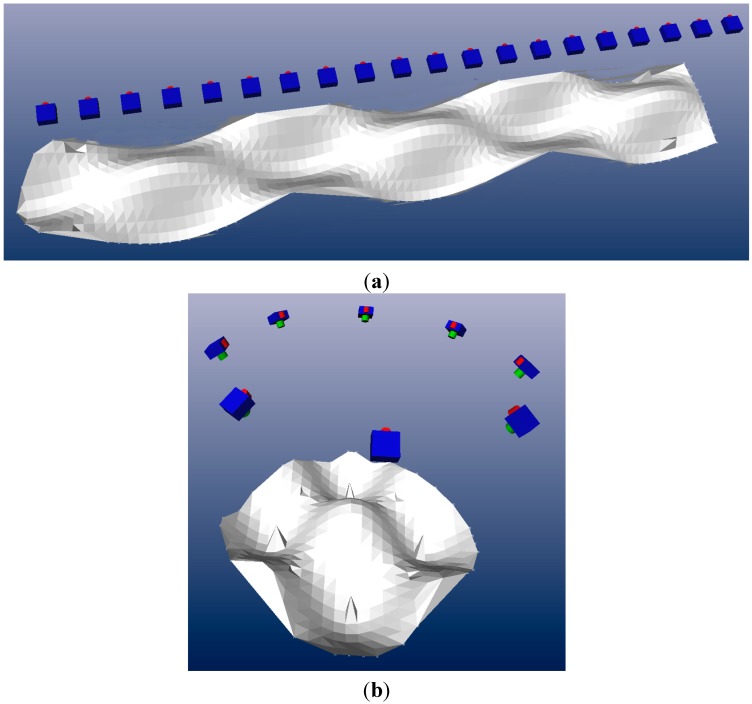
The two object shapes, including a typical camera network used as the basis for the numerical simulations: (**a**) single normal strip; (**b**) convergent circular block.

**Figure 5:**
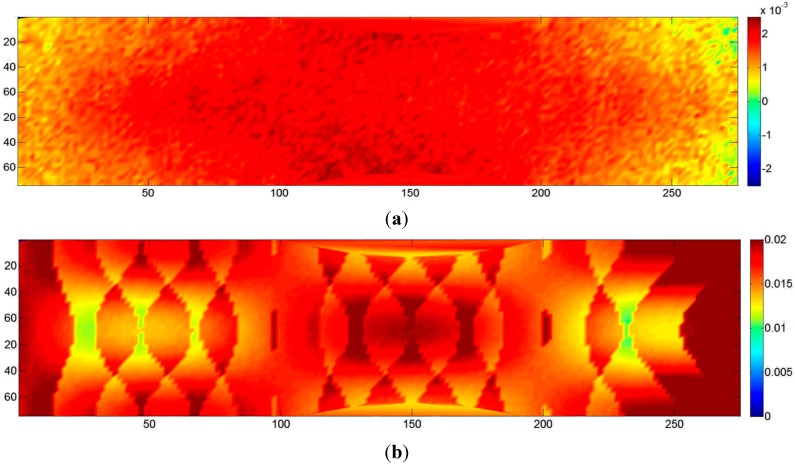
Case 1-A: single strip with four GCP (two on each end), 80% overlap, pre-calibrated. (**a**) Colour map of the mean error; (**b**) colour map of the standard deviation of the error.

Two object shapes were considered: the first (Figure 4a) is an elongated sinusoidal shape similar to the berm surface with an amplitude up to 20% of the scene depth; the second (Figure 4b) is very similar, but with a more compact domain. The objects and the related camera network are based on the setup presented in Section 2. Note that the camera network actually used in a particular case might slightly differ from the one shown in Figure 5. A Gaussian noise of 1% of the scene depth was added to the surfaces in order to add some more realistic irregularity to the objects. The measurement unit of the object space coordinates is directly related to the measurement unit of the error. In order to keep it general, both are chosen to be dimensionless and expressed in units (u). Note that u can be replaced with any unit. In the subsequent sections, the specific features of each case study are described in detail and the main results are discussed.

### 3.1. Case 1: Single Normal Strip

One of the most common form of image block geometry is represented by a single pseudo-normal strip of images (*i.e.*, with the image plane maintained parallel to itself along the sequence). In order to obtain the maximum uniformity in terms of precision, image scale and point identification, the distance between the camera and the object as well as the base length between adjacent images are constant within the block. The base length is usually chosen so that the overlap between consecutive images is between 60% (as in traditional aerial surveys) and 80% (more common in close-range digital camera surveys where the higher number of images generally improves the rigidity and stability of the block). 

The potential weakness of this configuration is two-fold. On the one hand, if a few GCP are provided in long strips, the error propagation can produce block deformation. In such cases, the object shape is locally still rather accurate, but the object is translated and rotated with respect to its correct position. On the other hand, if the user does not provide a set of calibrated IO parameters and, hence, relies on the self-calibration procedure implemented in the software, the outcome of the procedure might not be as desired. As shown by photogrammetry textbooks [14], such geometric configurations suffer from high correlation between IO and EO parameters (in particular, between the perspective centre height and the focal length) and become numerically ill-conditioned if the object is planar, regardless of the number and distribution of GCP. In particular, as far as a collinearity equation residual minimization scheme is implemented (all BBA routines use a least squares approach), the principal distances (PDs) and the distances between the object and the camera stations are often estimated as longer than the actual ones. This produces a non-uniform scaling of all of the tie points in the depth direction.

The MC simulation depicts quite clearly both issues. In the simulation, a sequence of 15 images, taken with 35-mm optics, a pixel size of 6 μm, full frame, with 80% overlap at 100 u distance from the object is considered. A total of about 10,000 tie points were considered, simulating an SfM orientation procedure with 0.5-pixel precision of the image coordinates. Equation (1) gives a theoretical accuracy of the object *z* coordinate of 0.06 u at 80% and of 0.03 u at 60% overlap. Just four coplanar GCP, two at each end of the strip, were considered. The GCP basically define the object reference system. They are not sufficient to effectively prevent the strip deformation. Additionally, being coplanar, they do not control the correlation between PD and EO. 

For Case 1-A, the camera is first calibrated before each MC simulation, and then, the single-strip block is adjusted using the updated parameters. The goal is to investigate the block behaviour for cases where the block adjustment is performed with fixed and calibrated interior and distortion parameters. The block used for calibration is configured as good photogrammetric practice suggests (see, for instance [27]): a highly redundant hemispherical block with multiple exposures at each station, with orthogonal roll angles to reduce IO and EO parameter correlation. Artificial targets are supposed to be used; hence a 0.2-pixel image point precision is considered. Thus, a new set of calibration parameters is estimated for every MC iteration and applied to the single-strip block adjustment of a new set of image coordinates. Finally, an automatic comparison between the true and estimated location of the object points is performed.

Figure 5a shows the actual deformation of the block, mapping the mean error evaluated in 1,000 MC iterations, while Figure 5b shows the corresponding standard deviations. At the start and at the end of the strip, where the tie points are visible in just two images, the mean standard deviation is 78 mu (30% poorer than the prediction of the theoretical model). For the central part of the block, where each tie point is seen in five images, the mean standard deviation is 17 mu, a value quite consistent with Equation (1), corrected with a factor $\frac{1}{\sqrt{k}}$ (with *k* = 5) to account for multi-ray intersections. It can be concluded that the pre-calibration uncertainty causes errors in the object coordinates of the same order of magnitude as the expected standard errors. Nevertheless, the error propagation models in Equations (1) and (2) do not consider the global image block deformation. The central part of the strip (*i.e.*, the area farthest from the GCP) has a deformation of 4–5 mu, and this is not negligible, since the standard deviation in these areas is consistently small.

**Figure 6:**
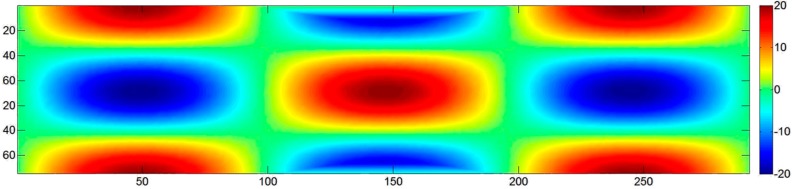
Case 1-B: single strip with four GCP (two on each end), 80% overlap, with self-calibration unbounded. Colour map of the mean error (errors are correlated to the point elevation and reach 20 u (units)).

Case 1-B consists of running the same single-strip simulation with self-calibration. The estimation of the IO parameters in the BBA leads, as expected, to unreliable results. CALGE estimates a focal length of 70 mm and a distance from the object of exactly 200 u for each MC iteration (the two parameters are 100% correlated). The *z* scale factor is two, and the object is scaled accordingly with errors up to 20 u (Figure 6). To overcome the problem, a pseudo-observation can be used to control the maximum change to the initial value of the PD. However, the system increases the PD to the maximum allowed value by the weight of the pseudo-observation equation. In other words, the numerical instability of the system is not solved, and the final value is constrained by a “magic” number that limits the *z* scale factor variation up to a certain threshold. In fact, the results are still unreliable.

To check if such behaviour is due to the particular solution routine in CALGE, the BBA was also run with other software programs. DBAT and PhotoModeler allow importing the very same image coordinates. Both packages exited with the warning of a singular normal equation system. As it is not possible to import image coordinates in PhotoScan, 15 synthetic images, taken in a normal case configuration, of the same object were generated using the 3D modelling software Blender [28] package and then imported. The software estimated the IO parameters in a self-calibration BBA, producing exactly the same results as CALGE (*i.e.*, a 70-mm focal length, with a distance from the object two-times higher than the correct one).

### 3.2. Case 2: Influence of Autofocus

Out-of-focus and depth of field issues generally arise for shooting distances less than 1 m. In such cases, a decision must be made on whether to maintain the same PD for each image (*i.e.*, disabling the autofocus) and accepting that not all images are perfectly focused, or to focus each time (manually or using the autofocus), accepting that the PD of the camera is not the same for the whole block. Usually, the latter is chosen, because blurred images make the homologous point identification harder and less precise. In that case, another decision must be taken for the BBA: if it is assumed that the PD variation is negligible, a sort of mean IO parameter set will be estimated; otherwise, a different focal length should be estimated for every image using a self-calibration approach.

In this case, the MC simulations were performed on a block made of a series of eight convergent images evenly distributed on a circle at about a 100-u distance from the object of Figure 4b. The tie point multiplicity varies from eight in the centre to three along the border of the object. Each image has its own focal length, derived adding a Gaussian noise to a mean value of 35 mm. The standard deviation σ of the Gaussian distribution was computed acquiring some images of a small object, fixing the focus ring of the camera and calibrating the camera every time. The results showed that σ = 0.1 mm is a realistic value and is hence used in the simulations.

**Figure 7:**
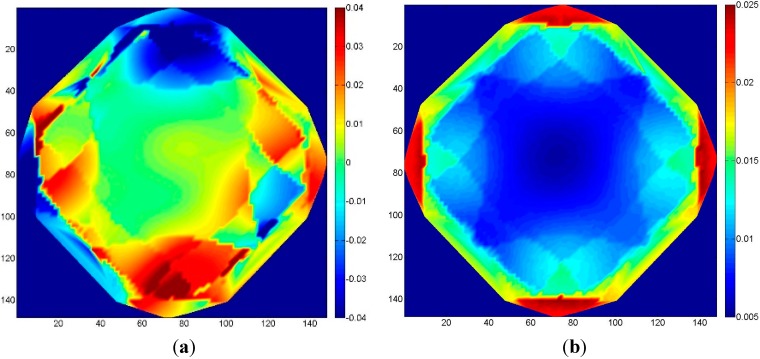
Case 2-A: the effect of neglecting focal length changes due to the use of autofocus with eight convergent images. (**a**) Mean error; (**b**) standard deviation of the error.

Considering a fixed PD (Case 2-A) produced the results shown in Figure 7, where the mean error and the corresponding standard deviation are depicted in Figure 7a,b respectively. The results indicate that at the object border, where just two or three images overlap, a much higher deformation occurs when compared to the central part, where all eight images concur to define the position of the object points. As far as the variation of the standard deviations is concerned (*i.e.*, how much the DSM should appear noisy), the wrong focal length seems not to produce relevant effects. In addition, the distribution of the standard deviation is symmetrical, with lower values in the central region of the object where tie point multiplicity is higher and with more noisy results in the outer parts where multiplicity is lower.

Even if the image block of Case 2-A is more rigid than the strip considered in Section 3.1, as far as self-calibration procedures are concerned, high correlation between parameters should be expected. In particular, the latter should be expected between focal length and principal point coordinates, unless the camera is sometimes rotated along its optical axis. However, when looking at the correlation matrix between the IO parameters, the correlations can be avoided by fixing one of the two parameters (usually the principal point coordinates). This latter approach was taken into account in the following simulation (Case 2-B).

**Figure 8:**
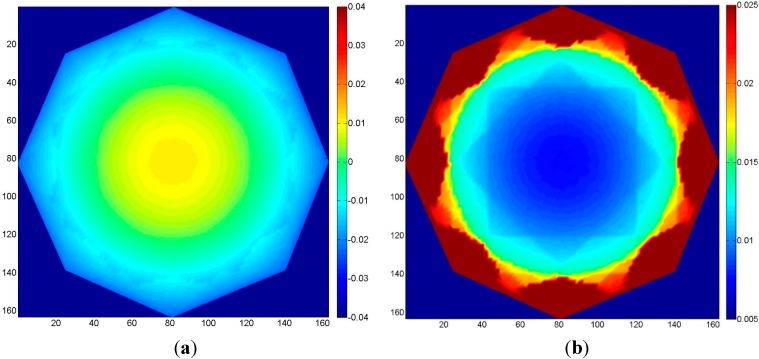
Case 2-B: eight convergent images; errors with camera the principal distances (PDs) estimated in the bundle block adjustment (BBA) (self-calibration). (**a**) Mean error; (**b**) standard deviation of the error.

The mean values of the errors (*i.e.*, object deformation) are shown in Figure 8a. It can be seen that in this case, the error distribution is symmetrical, with values about three-times lower than with the single PD simulation. However, the standard deviations are up to two-times higher (Figure 8b). In fact, the use of the self-calibration procedure removes any correlation effect between the parameters. This can lead to a more uniform distribution of the error (the model is less prone to localized deformation). Nevertheless, it can produce quantitatively larger errors: computing the median of root mean square (RMS) of the differences in the two cases, the calibrated scenario shows an error of 0.016 u, while the self-calibrated one shows an error of 0.021 u.

### 3.3. Case 3: Number of GCP

In Equations (1) and (2), the number and quality of the GCP (or, in general, of the ground control) are not taken into account. It is assumed that the GCP distribution is such that the rigidity of the block is guaranteed. While in airborne photogrammetry, the GCP layout is well defined, in close-range photogrammetry, the distribution of GCP is often strictly connected to the specific application. It is not always ensured that a rigid control structure can be provided for the survey. For instance, the central part of the berm in the full-scale experiment (Section 2.1) has no GCP, because they would be destroyed by the truck during the experiment. Moreover, at certain scales, it is difficult to provide GCP with an accuracy significantly better than the photogrammetric measurements expressed at the object scale. To test such effects, different simulations are performed over the object of Figure 4a, surveyed with a single strip. The overlap is varied from 60% (10 images) to 80% (20 images), while 4, 8 or 16 GCP are used to control the strip. In the case with four GCP (Case 3-A and 3-D), a pair is placed at each end of the block. In the other two cases, they are uniformly distributed in pairs along the strip.

Since block deformation occurs randomly at each MC iteration, the distribution of the deviation of each point should be used to assess the error in each simulation. However, to show the block deformation due to GCP number and distribution, the error component due to the collimation error is filtered. In other words, the error of the standard deviations measured comparing each point with the reference (error-free) 3D model is influenced by the collimation error and by the block deformation. Considering the high number of MC iterations, first the standard deviation error of a non-deformable image block is computed (*i.e.*, with EO parameters fixed at true values), and then, it is subtracted from the results of the simulations to assess the deviation due to block deformation dependent on the number of GCP.

**Figure 9:**
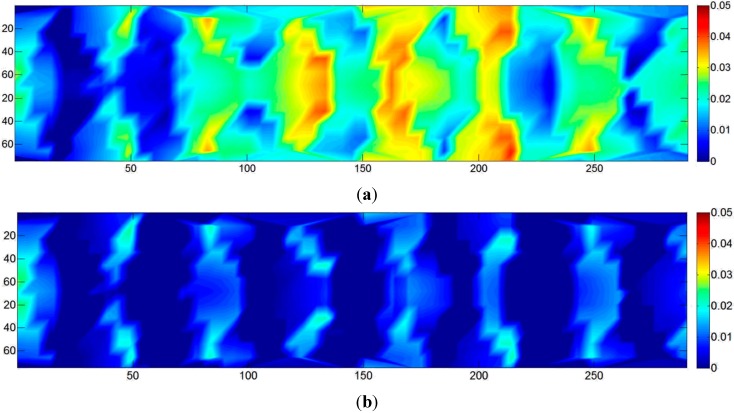
Case 3: standard deviation of the error with 60% overlap along a single strip as a function of the number of GCP: (**a**) Four GCP (Case 3-A); (**b**) eight GCP (Case 3-B).

Figure 9a,b shows the results for four and eight GCP, respectively, with 60% overlap: the first case with four GCP presents an evident deformation in the central part of the block. Here, the order of the magnitude of the block deformation error is approximately two-times higher than the standard deviation (approximately 0.02 u) of the collimation errors in the same area. On the contrary, with eight GCP (and even more in the case of 16 GCP), the block deformation is negligible with respect to the collimation standard deviation (Figure 9b).

**Figure 10:**
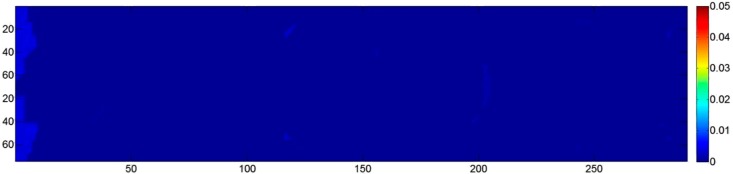
Case 3-D: standard deviation of the error with 80% overlap with four GCP.

Figure 10 shows the results of Case 3-D (four GCP) with 80% overlap. The high redundancy of ground point collimation and the increased rigidity given by the larger overlap effectively control the block deformation. This aspect, nonetheless, should be further investigated. Appropriately distributed and accurate GCP can assure an accurate block orientation. However, an increase in image overlap does not in itself imply a reduction of the number of GCP, especially if the block geometry and the distribution of tie points are not carefully taken into account.

### 3.4. Case 4: Quality of GCP

To test the influence of the quality of the GCP (*i.e.*, of their accuracy), a circular image sequence was used, similar to the one in Section 3.2. Eight GCP were evenly distributed on the edge of the object, and a random noise was applied to the 3D coordinates of these points at each MC iteration in order to simulate a coordinate error. The amount of error was related to the ground sampling distance (GSD) of a normal-case sequence at the same distance from the object, which corresponds to about 17 mu. Four MC simulations were performed, each time with a different standard deviation of the error on the GCP: 10-, 5-, 1- and 0.5-times the GSD.

**Figure 11:**
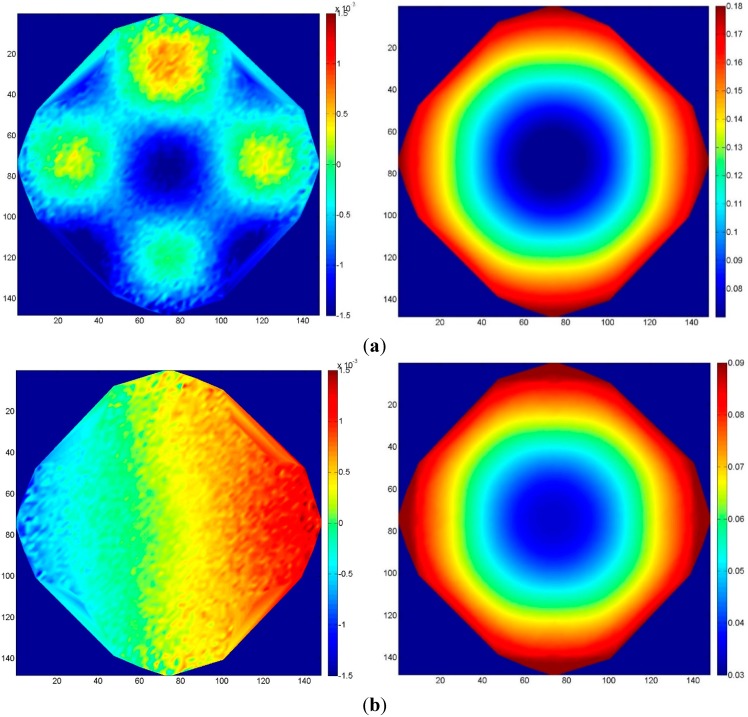

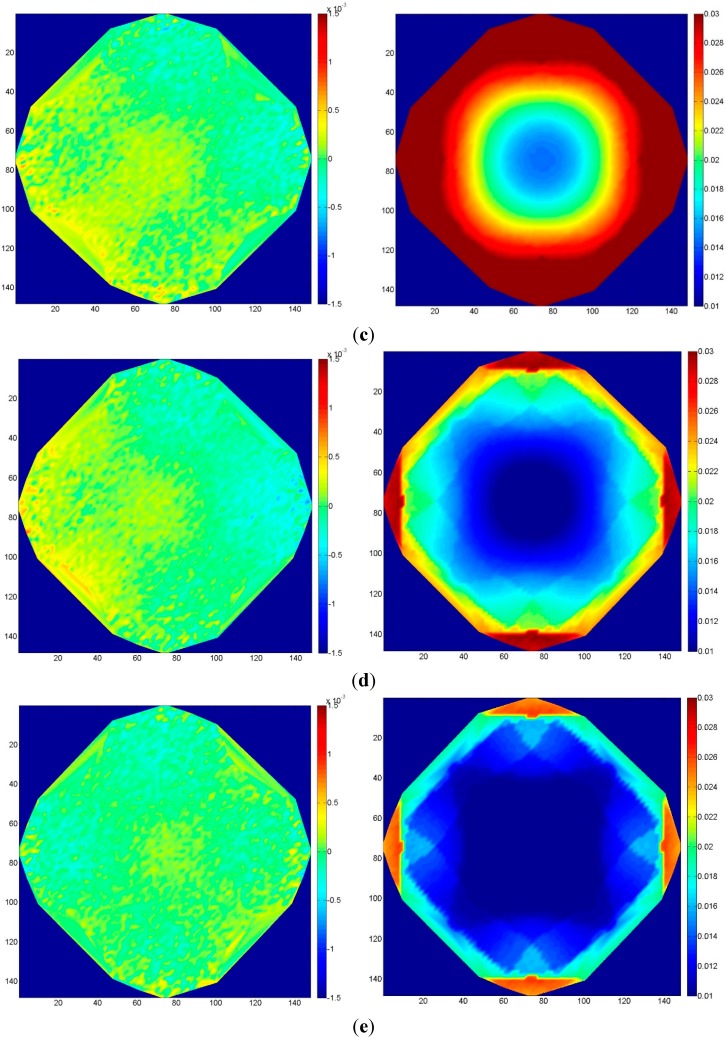
Case 4, circular block: errors due to varying GCP accuracy. Left column: mean error; right column: standard deviation of the error. From top to bottom, the GCP accuracy, expressed in terms of GSD, improves. (**a**) 10 GSD; (**b**) 5 GSD; (**c**) 2 GSD; (**d**) 1 GSD; (**e**) 0.5 GSD.

The results are summarized in Figure 11. From Figure 11a to Figure 11e, the accuracy of the GCP improves from 10 GSD to 0.5 GSD. The maximum standard deviation decreases from 0.22 u to 0.035 u. The results indicate that the precision of the GCP should be at least of the same order of magnitude of the GSD (a pixel image coordinate precision of 0.5 is assumed) to ensure that no systematic deformation and/or error propagation on the tie point coordinates arise.

### 3.5. Case 5: GCP to Tie Point Ratio

The last scenario investigated considers the influence of the ratio between the number of tie points and the GCP. Generally, least squares routines rescale the weight of the tie point image coordinates (collinearity equations) with respect to the weight associated with the coordinates of the GCP (usually implemented by means of a pseudo-observation of the coordinates). If the weight is not scaled according to the number of tie points, which can be very high in an SfM image block, the GCP lose importance in the final residual least squares analysis, and they are not strong enough to constrain the block appropriately. In the following, the same block as in Section 3.4 with eight GCP was considered.

**Figure 12:**
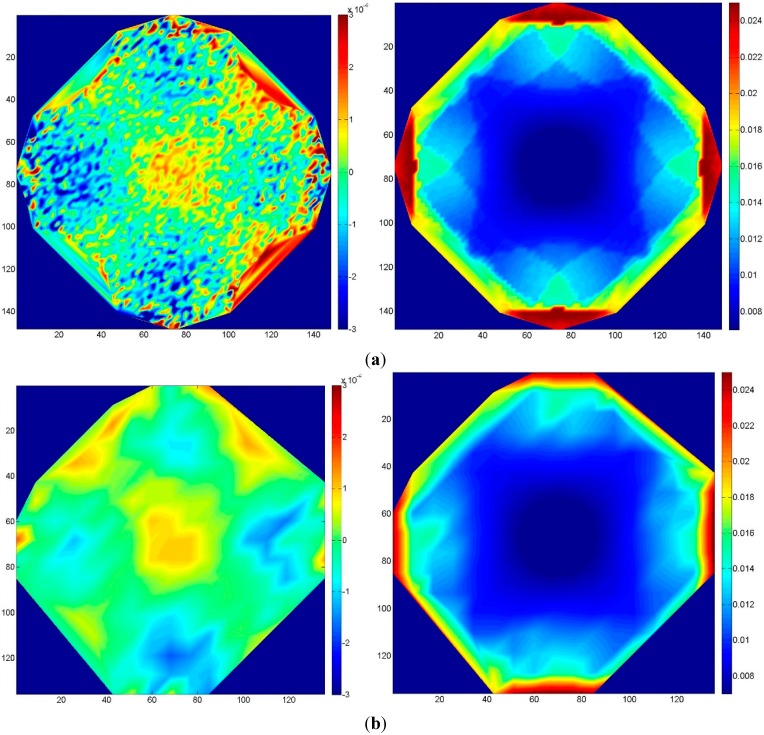
Case 5: errors due to using a varying number of tie points. Left column: mean error; right column: standard deviation of the error. (**a**) 10,000 tie points; (**b**) 500 tie points.

As shown in Figure 12, the results obtained using a few tie points does not significantly differ from the ones obtained with many. In other words, considering the precision of both, a common SfM software package and the GCP, hardly any difference is noticeable when using many tie points (as usually an SfM routine does) or just a few. As reported by Stamatopoulos and Fraser [15], the problem could be much more serious if the deformation is assessed using a certain number of artificial targets. In that case, the image coordinate accuracy of the points extracted by the SfM on natural features would probably be less precise compared to the ones extracted by image matching on the artificial target, and the stochastic model would probably have produced some unwanted deformation.

## 4. Conclusions and Recommendations

The progress in automatic image orientation and surface reconstruction has made available easy-to-use, turnkey software that is making photogrammetry increasingly appealing also to untrained or inexperienced users. The documentation of the results provided by these programs is often lacking in detail, giving the feeling that everything is always right. Block design, however, should not be forgotten, nor should there be the need for internal and external checking of the results. Starting from discrepancies found between the theoretical and empirical precision of full-scale and laboratory surveys, a series of MC simulations on two common block types has been performed. The results show that self-calibration should be used carefully and that its uncertainty might affect object coordinates significantly. Even in convergent blocks, when using autofocus at short range, it is not evident that the use of individual self-calibration of each image (opposed to an average calibration) is more convenient, as far as the variations of the PD are small. When looking at the influence of the number of GCP, the results indicate that there might be an optimum number. Indeed, in the single normal-case sequence, an increase of the GCP from eight to 16 did not improve the results. On the one hand, the results clearly show that GCP uncertainty must at least match the image GSD: with very small object sizes (as in the case of small-scale experiments) or with very small GSD (as with unmanned aerial systems), this is not always easy to achieve. On the other hand, the effectiveness of the GCP constraints when considering a high number of tie points showed a small influence in the case of a convergent circular block.

To summarize, full-scale and laboratory surveys need to be designed accounting for the effect of a number of parameters or disturbances. As shown in this paper, simulations provide a framework and guidelines for such a design, a stage that should never be underestimated. Especially when the specific conditions of a given experiment might introduce constraints on the actual implementation of the photogrammetric network, making the fulfilment of an ideal setup just partial, a sensitivity analysis is possible and recommendable. As a rule of thumb, the design and implementation of laboratory-scale experiments are more critical than full-scale experiments, since the required accuracy is generally higher, and hence, the effects of unaccounted factors are more likely to influence the accuracy of the results.
